# Effekt der COVID-19-Pandemie und des Lockdowns auf die Inzidenz von Herzinfarktpatienten in Deutschland – Ergebnisse einer Metaanalyse

**DOI:** 10.1007/s12181-021-00479-4

**Published:** 2021-06-21

**Authors:** Manuel Rattka, Jens Dreyhaupt, Claudia Winsauer, Lina Stuhler, Michael Baumhardt, Wolfgang Rottbauer, Armin Imhof

**Affiliations:** 1 Klinik für Innere Medizin II, Kardiologie, Pneumologie, Angiologie, Intensivmedizin, Universitätsklinikum Ulm, Albert Einstein Allee 23, 89081 Ulm, Deutschland; 2 Institut für Epidemiologie und Medizinische Biometrie, Universität Ulm, Ulm, Deutschland

**Keywords:** COVID, Herzinfarkt, Inzidenz, Deutschland, Epidemiologie, COVID, Myocardial infarction, Incidence, Germany, Epidemiology

## Abstract

**Hintergrund:**

Mit dem Beginn der COVID-19-Pandemie wurde weltweit über das Phänomen der rückläufigen Einweisungen von Herzinfarktpatienten berichtet. In dieser Metaanalyse wird die Häufigkeit der Vorstellungen von STEMI- und NSTEMI-Patienten in Deutschland während und vor der COVID-19-Pandemie analysiert.

**Methoden:**

Es erfolgte eine selektive Literaturrecherche mit den Suchbegriffen „COVID“ und „myocardial infarction“ oder „STEMI“ oder „NSTEMI“ und „Germany“ in PubMed, Web of Science und Embase.

**Ergebnisse:**

Basierend auf unserer Suchstrategie, konnten aus 40 identifizierten Studien 5 in unsere Metaanalyse aufgenommen werden. Diese ergab, dass die Häufigkeit der Krankenhauseinweisungen von Patienten mit akutem Myokardinfarkt in Deutschland während der Pandemie signifikant reduziert war (Inzidenzratenverhältnis [Incidence Rate Ratio, IRR] = 0,849, 95%-Konfidenzintervall: 0,827–0,872). Dies traf sowohl auf Patienten mit STEMI (IRR = 0,875, 95%-Konfidenzintervall: 0,837–0,914) als auch auf Patienten mit NSTEMI (IRR = 0,760, 95%-Konfidenzintervall: 0,633–0,911) zu.

**Schlussfolgerung:**

In der vorliegenden Metaanalyse konnten wir zeigen, dass auch in Deutschland während der COVID-19-Pandemie die Häufigkeit der Krankenhauseinweisungen von Herzinfarktpatienten drastisch rückläufig war. Während der noch andauernden Pandemie ist es essenziell, die Bevölkerung weiterhin über die Symptome eines Herzinfarktes und die Dringlichkeit der akuten medizinischen Versorgung zu informieren und aufzuklären, um einer potenziellen Gefährdung von Herzinfarktpatienten vorzubeugen.

**Zusatzmaterial online:**

Die Online-Version dieses Beitrags (10.1007/s12181-021-00479-4) enthält eine ausführlichere Darstellung der Vorgehensweise, Methoden und Limitationen. Beitrag und Zusatzmaterial stehen Ihnen auf www.springermedizin.de zur Verfügung. Bitte geben Sie dort den Beitragstitel in die Suche ein, das Zusatzmaterial finden Sie beim Beitrag unter „Ergänzende Inhalte“.
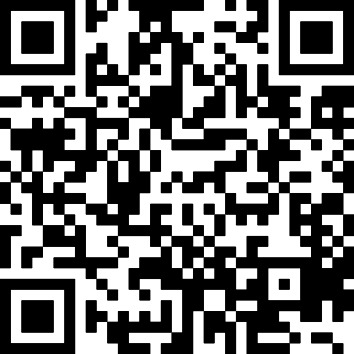

## Abstract (en)

**Background:**

Since the beginning of the COVID-19 pandemic there have been observations from around the world reporting on the phenomenon of a reduction in numbers of patients with myocardial infarction. In this meta-analysis the incidences of STEMI and NSTEMI patient admissions in Germany during and before the COVID-19 pandemic were analyzed.

**Methods:**

A comprehensive literature search was performed through PubMed, Embase and Web of Science using combinations of the keywords “COVID”, and “myocardial infarction”, or “STEMI”, or “NSTEMI”, and “Germany”.

**Results:**

Based on our search strategy out of 40 identified studies 5 were included in the meta-analysis. The hospital admission rates of patients with acute myocardial infarction in Germany significantly declined during the pandemic (incidence rate ratio, IRR = 0.849, 95% confidence interval 0.827–0.872). This was the same for patients with STEMI (IRR = 0.875, 95% confidence interval 0.837–0.914) and NSTEMI (IRR = 0.760, 95% confidence interval 0.633–0.911).

**Discussion:**

In this meta-analysis we could show that in Germany the frequency of hospital admissions of patients with myocardial infarction significantly declined during the COVID-19 pandemic. Consequently, during the ongoing pandemic it is crucial to continue informing and educating the public on the symptoms of a heart attack and the necessity of timely medical care to avoid potential endangerment of patients with myocardial infarction.

Seit Beginn des SARS-CoV-2(Schweres-akutes-Atemwegssyndrom-Coronavirus Typ 2 [severe acute respiratory syndrome coronavirus type 2])-Ausbruches mehren sich die Hinweise, dass die Pandemie das Gesundheitsverhalten und die medizinische Versorgung der Bevölkerung negativ beeinflusst [3, 8]. So wurde gezeigt, dass in Deutschland die Fallzahlen für diverse Bereiche der Krankenhausversorgung zu Beginn der Pandemie rückläufig waren [4]. Mutmaßlich lässt sich dies auf die notwendige Erhöhung der Kapazitäten der Kliniken zur Versorgung von COVID-19-(Coronavirus-Krankheit-2019 [coronavirus disease 2019])-Infizierten und die damit verbundene Reduktion von elektiven Behandlungen zum einen und auf ein mit der Angst vor einer innerklinischen Infektion mit SARS-CoV‑2 begründetes Vermeidungsverhalten von Patienten zum anderen zurückführen [4, 5]. Wir berichten hier über das Ausmaß des Effektes der COVID-19-Pandemie auf die notfallmäßige Vorstellung von Herzinfarktpatienten in Deutschland.

## Methode

Es erfolgte eine selektive Literatursuche in PubMed, Web of Science und Embase mit den Suchbegriffen „COVID“ und „myocardial infarction“ oder „STEMI“ oder „NSTEMI“ und „Germany“. Eine ausführlichere Darstellung der Vorgehensweise und Methoden sowie die Limitationen befinden sich im ergänzenden Material.

## Ergebnisse

Basierend auf unserer Suchstrategie wurden 40 Studien identifiziert, davon 13 Duplikate. Von den verbleibenden Studien waren 20 Studien nicht mit der Fragestellung assoziiert. Von den 7 Studien, welche über die Versorgung von Herzinfarktpatienten in Deutschland berichteten, ließen sich aus 5 Studien Daten hinsichtlich der Inzidenz von Krankenhauseinweisungen von Patienten mit ST-Strecken-Hebungsinfarkt (STEMI) und/oder Nicht-ST-Strecken-Hebungsinfarkt (NSTEMI) extrahieren. Diese wurden in die Metaanalyse inkludiert [1, 5, 9–11]. Eine Übersicht der eingeschlossenen Studien ist der Tab. 1 zu entnehmen.

**Table 1:** 

Studie	Herkunft	Beobachtungszeitraum	Patienten Gesamt (*n*)	STEMI-Patienten (*n*)	NSTEMI-Patienten (*n*)
Dreger et al. [1]	Berlin	*COVID-19*	207	105	102
		09.03.–05.04.2020			
		*Prä-COVID-19*	762	341	421
		13.03.–09.04.2017			
		12.03.–08.04.2018			
		11.03.–07.04.2019			
Rattka et al. [5]	Ulm	*COVID-19*	32	16	16
		21.03.–20.04.2020			
		*Prä-COVID-19*	144	40	104
		21.03.–20.04.2017/18/19			
Scholz et al. [9]	FITT-STEMI-Studie	*COVID-19*	N/A	387	N/A
		01.03.–31.03.2020			
		*Prä-COVID-19*		1329	
		01.03.–31.03.2017/18/19			
Schwarz et al. [10]	Saarland	*COVID-19*	50	21	29
		02.03.–19.04.2020			
		*Prä-COVID-19*	63	28	35
		04.03.–21.04.2019			
Seiffert et al. [11]	BARMER Deutschland	*COVID-19*	9458	2940	6518
		01.01.–31.05.2020			
		*Prä-COVID-19*	11.032	3350	7682
		01.01.–31.05.2019			

*STEMI* ST-Strecken-Hebungsinfarkt, *NSTEMI* Nicht-ST-Strecken-Hebungsinfarkt, *N/A* nicht vorhanden

**Figure 1:**
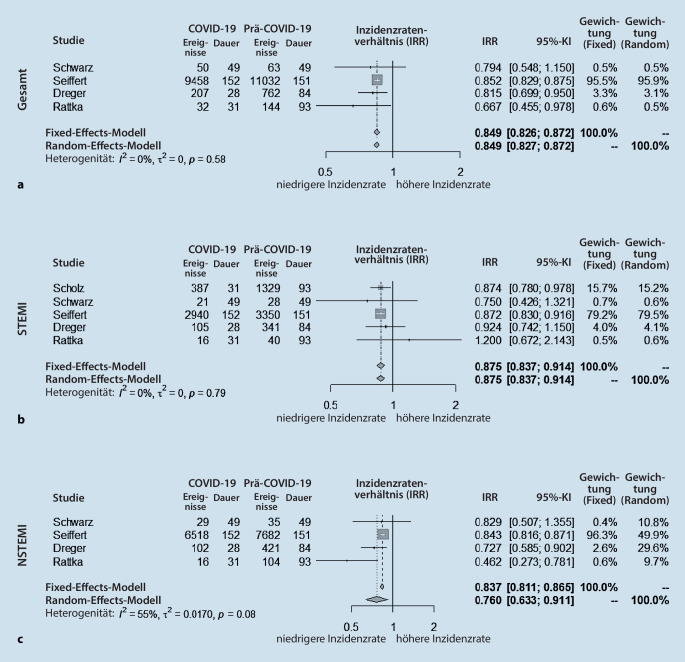

**Figure 2:**
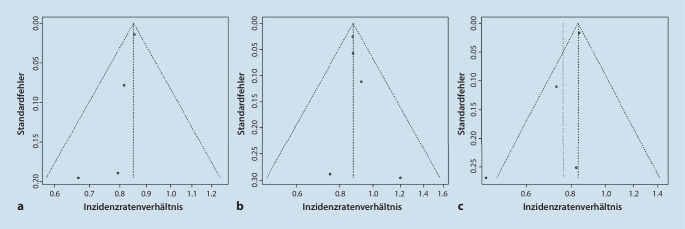

Insgesamt wurden in unsere Metaanalyse 23.464 Herzinfarktpatienten aus den identifizierten Studien eingeschlossen. Hiervon wurden 10.134 Patienten während der COVID-19-Pandemie (COVID-19) und 13.330 Patienten vor der COVID-19-Pandemie (Prä-COVID-19) vorstellig. Die Metaanalyse der Inzidenz ergab, dass die Häufigkeit der Krankenhauseinweisungen von Patienten mit akutem Myokardinfarkt in der COVID-19-Gruppe gegenüber der Prä-COVID-19-Gruppe signifikant geringer war (Inzidenzratenverhältnis [Incidence Rate Ratio, IRR] = 0,849, 95 %-Konfidenzintervall: 0,827‑0,872, I^2^ = 0 %; Abb. 1a). Die Subanalyse zeigte, dass sowohl die Anzahl an Einweisungen von Patienten mit Nicht-ST-Stecken-Hebungsinfarkt (NSTEMI) (IRR = 0,760, 95 %-Konfidenzintervall: 0,633–0,911, I^2^ = 55 %; Abb. 1c) als auch die von Patienten mit ST-Strecken-Hebungsinfarkt (STEMI) (IRR = 0,875, 95 %-Konfidenzintervall: 0,837–0,914, I^2^ = 0 %; Abb. 1b) signifikant sank. Die jeweils zugehörigen Funnel-Plots sind in Abb. 2 aufgeführt.

## Diskussion

In der vorliegenden Metaanalyse konnten wir zeigen, dass die Inzidenz der Zuweisungen von Patienten mit akutem Herzinfarkt in Deutschland während der COVID-19-Pandemie signifikant sank. Sowohl die Inzidenz für Patienten mit STEMI als auch NSTEMI zeigte sich signifikant reduziert.

Bereits kurz nach Auftreten der ersten SARS-CoV-2-Infektionen wurde über einen potenziellen ungünstigen Effekt der Pandemie auf die medizinische Versorgung spekuliert [3, 8]. Es wurde zur Diskussion gestellt, ob ggf. die Angst vor einer SARS-CoV-2-Infektion im Krankenhaus, eine Fehldeutung von Symptomen durch Patienten und medizinisches Personal oder auch altruistisches Verhalten, um das Gesundheitssystem möglichst nicht zu überlasten, zur Folge haben, dass Patienten trotz des Vorliegens von Symptomen nicht oder nur verzögert medizinische Hilfe aufsuchten [5]. Dies ist insbesondere für Patienten mit einem akuten Koronarsyndrom relevant, für die eine verlängerte Ischämiezeit eine Verschlechterung der Prognose zur Folge hat [2]. In unserer Metaanalyse konnten wir zeigen, dass auch in Deutschland, welches im internationalen Vergleich zu Beginn des COVID-19-Ausbruches verhältnismäßig weniger schwer von der Pandemie getroffen wurde, signifikant weniger Patienten mit STEMI oder NSTEMI vorstellig wurden als vor der Pandemie. Dies deckt sich mit den Beobachtungen aus schwerer betroffenen Ländern wie Italien und China [7, 12]. Im Gegensatz zu diesen Ländern konnten für Deutschland bisher keine signifikant verlängerte Zeit von Symptombeginn bis zum ersten medizinischen Kontakt, keine erhöhten Komplikations- oder erhöhte intrahospitale Mortalitätsraten nachgewiesen werden [6, 7, 9, 11, 12]. Dies lässt vermuten, dass eine zeitnahe und adäquate Versorgung von Patienten mit akutem Herzinfarkt in Deutschland, trotz der erhöhten Anforderungen an das Gesundheitssystem während der Pandemie, aufrechterhalten werden konnte. Nichtsdestotrotz ist es essenziell, die Bevölkerung weiterhin über die Symptome eines Herzinfarktes und die Dringlichkeit der akuten medizinischen Versorgung zu informieren und aufzuklären, um so während der noch andauernden COVID-19-Pandemie und aktuell wiederkehrenden Maßnahmen der sozialen Distanzierung einer potenziellen Gefährdung von Herzinfarktpatienten vorzubeugen.

## Fazit für die Praxis

- Auch in Deutschland zeigte sich während der bisherigen Pandemie ein signifikanter Rückgang an Krankenhauseinweisungen von Patienten mit akutem Myokardinfarkt. Dies gilt sowohl für STEMI- als auch für NSTEMI-Patienten.
- Die aktuelle Studienlage lässt annehmen, dass im Gegensatz zu anderen Ländern in Deutschland bisher eine zeitnahe und adäquate medizinische Versorgung von Herzinfarktpatienten aufrechterhalten werden konnte.
- Nichtsdestotrotz ist es essenziell, die Bevölkerung insbesondere während der noch andauernden Pandemie mit wiederkehrenden Maßnahmen der sozialen Distanzierung weiterhin über die Anzeichen eines Herzinfarktes und die damit verbundene Dringlichkeit einer notfallmedizinischen Versorgung aufzuklären.

## Supplementary Information




