# Supplementary material for: Effect of the COVID-19 pandemic and lockdown on the incidence of myocardial infarction patients in Germany—Results of a meta-analysis
Source: Kardiologe. 2021 Jun 21;15(4):407–11. [Article in German] doi: 10.1007/s12181-021-00479-4 (PMC8215859; doi:10.1007/s12181-021-00479-4)
Supplement: Supplementary file 1 [file 12181_2021_479_MOESM1_ESM.docx]

**Zusatzmaterial**

**Electronic Supplementary Material (ESM)**

**Methoden**

**Datenquellen und Studienselektion**

Es erfolgte eine selektive Literaturrecherche in PubMed, Web of Science, und Embase mit den Suchbegriffen „COVID“ und „myocardial infarction“ oder „STEMI“ oder „NSTEMI“ und „Germany“. Gemäß unserer vorab definierten Kriterien wurden Publikationen mit Veröffentlichung zwischen Anfang Januar und Ende November 2020 berücksichtigt. Die jeweilige studienspezifische Definition der „Prä-COVID-19 Gruppe“ und der „COVID-19 Gruppe“ wurde berücksichtigt.

**Datenextraktion und Studienqualität**

Zwei Autoren führten die Literaturrecherche unabhängig voneinander durch und extrahierten die relevanten Daten mittels eines standardisierter Formulars. Im Falle von Unstimmigkeiten wurde ein dritter Autor hinzugezogen. Für unsere Metaanalyse wurden alle Studien berücksichtigt, welche zwischen Anfang Dezember 2019 und Ende November 2020 veröffentlich wurden, und die die Anzahl der Krankenhauseinweisungen von Patienten mit ST-Strecken Hebungsinfarkt (STEMI) und/oder Nicht-ST-Strecken Hebungsinfarkt (NSTEMI), in einer definierten Periode vor sowie während der COVID-19 Pandemie berichteten, und miteinander verglichen. Da die Metaanalyse Daten von bereits veröffentlichten Studien umfassten war kein Ethikvotum notwendig. Die Studie wurde unter Beachtung der PRISMA Leitlinien durchgeführt. Die Übersichtsarbeit wurde prospektiv auf PROSPERO registriert (CRD42020223051).

**Statistische Analyse**

Die Daten wurden in einer Microsoft Excel® Datei gesammelt und eine Metaanalyse, bezogen auf den binären Endpunkt der Krankenhauseinweisungen von Herzinfarktpatienten vor und während der COVID-19 Pandemie, zur Berechnung des Inzidenzratenverhältnisses (Incidence Rate Ratio, IRR) inklusive des 95% Konfidenzintervalls als Effektgröße durchgeführt. Das Modell mit zufälligen Effekten wurde zur Kombination der Ergebnisse der eingeschlossenen Studien verwendet. Dieses Modell wurde aufgrund der zum Teil zu erwartenden moderaten oder starken Heterogenität, sowie der konservativeren Schätzung, dem Modell mit festen Effekten gegenüber vorgezogen. Zur Berechnung des Effektmaßes wurde die Mantel-Haenszel Methode angewendet. Die Heterogenität zwischen den Studien wurde mit dem I2-Maß untersucht und der p-Wert des zugehörigen chi2 Tests angegeben (ein Grenzwert von <0.05 suggeriert Heterogenität). Für die graphische Darstellung der Ergebnisse der Metaanalyse wurde der Forest Plot verwendet, welcher sowohl die Ergebnisse der einzelnen eingeschlossenen Studien als auch das Gesamtergebnis visualisiert. Zudem wurden Funnel Plots erstellt, um das Risiko eines Publikationsbias zu evaluieren. Die Metaanalysen wurden mit dem R Paket „meta“ der Statistiksoftware R durchgeführt (Version 3.5.1). Ergebnisse mit einem zweiseitigen p Wert von <0.05 wurden als statistisch signifikant interpretiert.

**Limitationen**

Hier stellen wir die Ergebnisse einer prospektiv registrierten Metaanalyse dar, welche die Inzidenzraten von Herzinfarktpatienten, welche in Deutschland vor und während der COVID-19 Pandemie notfallmäßig eingewiesen wurden, vergleicht. Aufgrund des akuten Auftretens der COVID-19 Pandemie, sind bisher nur retrospektive Studien sowie Ergebnisse aus Registern veröffentlicht, die über die Anzahl der Einweisungen von Herzinfarktpatienten berichten. Folglich war es nicht möglich, in diese Metaanalyse randomisierte, prospektive Studien einzubeziehen, was die Aussagekraft limitiert. Weiterhin bestehen aufgrund der limitierten Anzahl verfügbarer Studien, sowie der unterschiedlichen Anzahl der jeweils eingeschlossenen Patienten, deutliche Unterschiede hinsichtlich der Gewichtung der einzelnen Studien. Dies stellt jedoch ein typisches Merkmal von Metaanalysen dar, und wird unter anderem dadurch berücksichtigt, dass auf das Paneldatenmodell mit zufälligen Effekten zur Berechnung der Signifikanz zurückgegriffen wurde. Bemerkenswerterweise weisen die Analysen insgesamt eine sehr geringe bis moderate Heterogenität auf, so dass wir annehmen mit dieser Arbeit einen validen Eindruck bezüglich der Inzidenzraten von Herzinfarktpatienten in Deutschland vor und während der COVID19 Pandemie geben zu können.
